# Digital health equity among older adult(s): a conceptual analysis

**DOI:** 10.3389/fpubh.2026.1891578

**Published:** 2026-07-06

**Authors:** Xinchi Guo, Jingmei Cao, Mingjiang Huang, Tongyu Tan, Haishan Quan, Chunyu Li, Xiangdan Shen

**Affiliations:** School of Nursing, Yanbian University, Yanji, Jilin, China

**Keywords:** concept analysis, digital health equity, healthy aging, nursing, older adults

## Abstract

**Aims:**

To analyze and clarify the conceptual connotation of digital health equity for the older adults, providing a reference for the future development of assessment tools and the formulation of related policies.

**Methods:**

Concept analysis was conducted using Rodgers' evolutionary method. A systematic search of CNKI, Wanfang Data, VIP Database, PubMed, Web of Science, Cochrane Library, CINAHL, and Embase was conducted from their inception to November 30, 2025

**Results:**

A total of 44 articles were included. The defining attributes of digital health equity among older adults are digital health resource equity, digital access capability equity, and digital cultural adaptation equity; antecedents include sociodemographic factors, individual factors, family factors, social factors, and technological factors; consequences include both positive consequences and negative consequences.

**Conclusion:**

Clarifying the concept of digital health equity for the older adults can provide ideas for the development of assessment tools and theoretical models, as well as a basis for policy formulation, technology design, and service optimization.

## 1 Introduction

With the accelerated global digitalization, new technologies characterized by informatization have propelled the traditional healthcare service system into a new stage of digital health development (1). Digital health encompasses tools and services that enhance health and facilitate disease management through technology. This includes mobile health, virtual care, remote monitoring, data exchange tools, and artificial intelligence (2, 3). Digital approaches can eliminate temporal and spatial barriers, allowing for precision, personalization, and universal accessibility in health management. They are important tools for reducing health disparities (4). Despite the rapid growth of digital health technologies, older adults still do not share equally in the benefits. Older adults often encounter operational difficulties, technical barriers, and privacy and security concerns when using basic digital tools such as smartphones, online appointment systems, and health codes (5, 6). Research shows that older adults accept and use these technologies at much lower rates than other age groups (7). The core issue of these phenomena is the rise of digital health inequity. Digital health inequality refers to the ongoing structural social inequalities that persist even with the widespread use of digital technologies. This results in systematic differences in access to, understanding of, and use of digital health resources among older adults. These disparities affect individuals across various socioeconomic, geographic, and educational backgrounds (8). This inequality limits older adults' access to health information and may worsen health inequities (4). Based on this, digital health equity among older adults has emerged as a key concept. It concerns not only the widespread adoption of technology but also whether the health outcomes generated by technology are equitable (9). There is currently considerable confusion surrounding the concept of digital health equity for older adults. Many related concepts frequently appear in the literature but lack clear definitions, thereby hindering the in-depth advancement of related research and the precise implementation of policies and services. Therefore, there is an urgent need to systematically clarify the concept and connotations of digital health equity for older adults. This study utilized Rodgers' (10) evolutionary concept analysis method to summarize and synthesize the concept of digital health equity for older adults. The aim is to provide a theoretical foundation and decision-making reference for developing assessment tools, formulating related policies, and optimizing technology and service models.

## 2 Methods

### 2.1 The process of concept analysis

Rodgers argued that the aim of concept analysis is to clarify concepts and their current usage, using their attributes as a foundation for further development. This process captures the dynamic changes that concepts experience over time and across different contexts (11). The specific steps included selecting digital health equity for older adults as the concept of interest; collecting data and determining the sample; examining the contexts in which the concept appeared and its alternative terms, and identifying related concepts and applications; defining its core attributes and components with precision; exploring in depth the antecedents influencing its realization and the consequences arising from it; constructing and analyzing model cases; and discussing the further development of digital health equity for older adults to clarify its theoretical positioning and practical value.

### 2.2 Data sources

Retrieval was performed using multiple databases, including PubMed, Web of Science, Cochrane, CINAHL, Embase, CNKI, VIP Database, and Wanfang. Based on the characteristics of different databases, Boolean logic was applied to search the titles and abstracts of articles, using terms such as “digital health equity,” “digital health,” “telemedicine,” and “mHealth” for retrieval. The retrieval period spanned from database inception to November 30, 2025. The specific search strategies are detailed in the Supplementary material.

The included literature focused on older adults and centered on digital health equity, encompassing Chinese- and English-language studies on the conceptual development of digital health equity, its defining attributes, antecedents and consequences, assessment tools, and typical cases. Conference papers, duplicate publications, and literature with inaccessible full texts were excluded.

**Figure 1:**
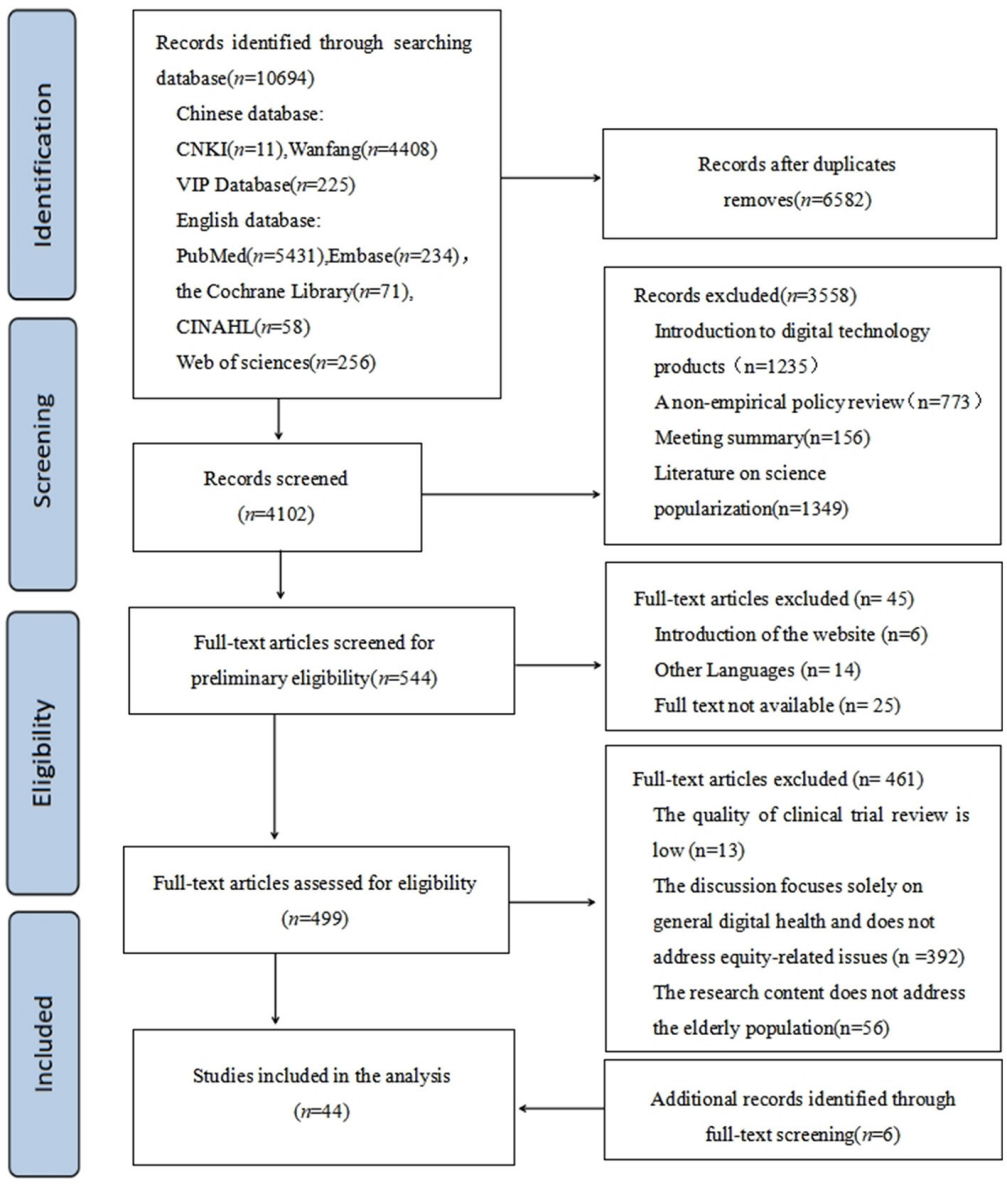
Flow diagram of literature search and selection.

Literature screening was conducted independently by two researchers, and any disagreements were resolved through discussion with a third researcher. A total of 10,694 articles were retrieved, of which 4,102 remained after deduplication using EndNote X9. Based on this, a screening of titles and abstracts was conducted for each article. Consequently, 3,558 articles were excluded because they consisted solely of introductions to digital products, policy overviews without empirical data, conference abstracts, or popular science reviews. This process left 544 articles for further analysis. Subsequently, the remaining 544 articles underwent a first-round screening. Of these, 45 articles were excluded, including 6 that introduced platform websites, 14 published in languages other than Chinese or English, and 25 for which the full text was unavailable. This process left 499 articles after the initial screening. After conducting a full-text review, a total of 461 articles were excluded. This included 13 articles identified as low-quality clinical trial reviews, 392 articles that discussed general digital health without addressing equity issues, and 56 articles whose research did not focus on older adults. Because some relevant studies were not captured in the initial search due to variations in titles or keywords, an additional six papers were identified through reference screening during the full-text review stage. These Supplementary material primarily addressed theoretical frameworks and key factors influencing digital health equity among older adults. In total, 44 eligible studies were included, comprising 7 Chinese-language studies and 37 English-language studies. To meet Rodgers' concept analysis method, the included literature had to account for more than 20% of the total body of literature or comprise at least 30 studies (11). A detailed flowchart of the literature search process is shown in Figure 1.

## 3 Results

### 3.1 Evolution of digital health equity among older adults

**Figure 2:**
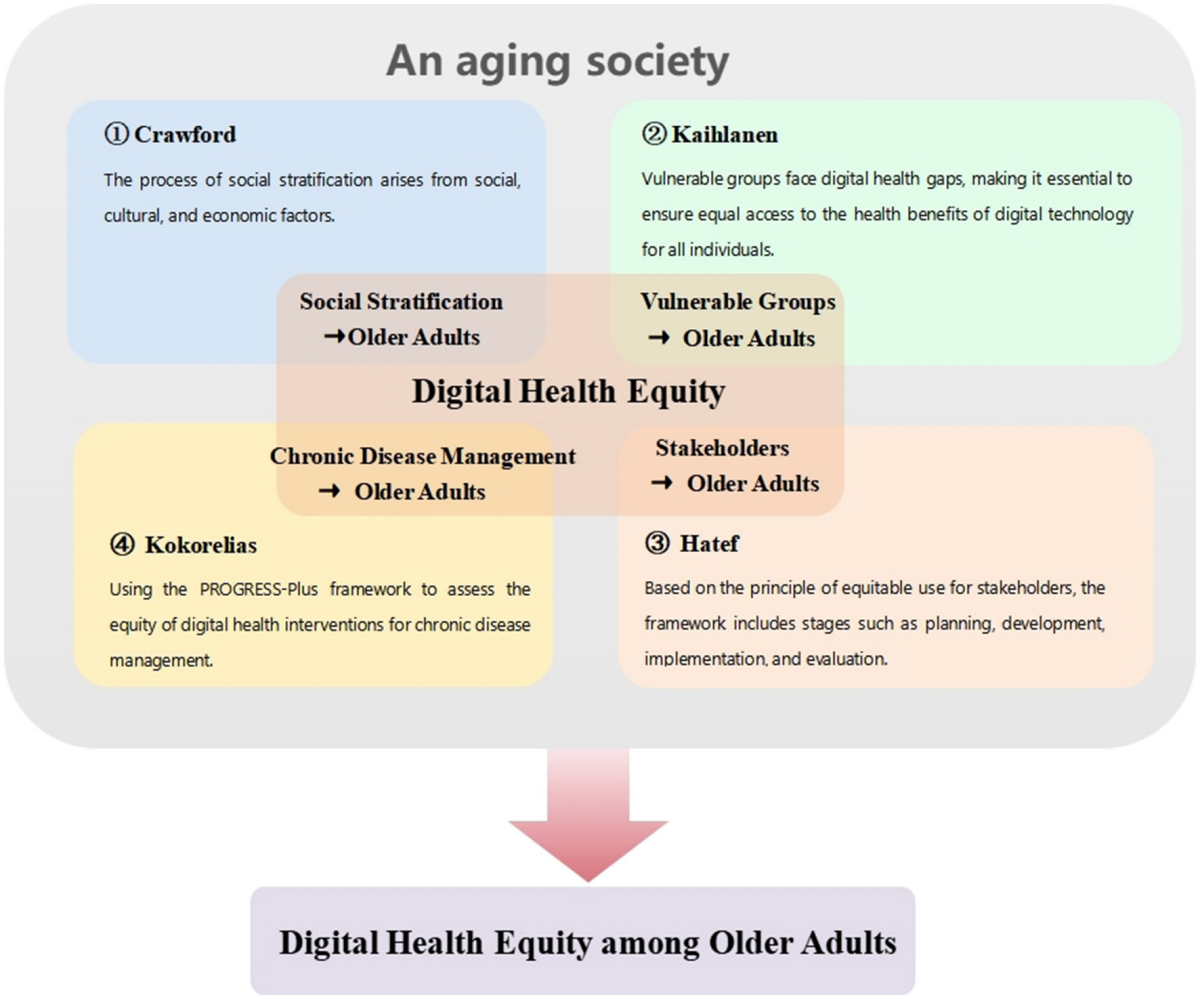
Evolution of the concept of digital health equity among older adults.

Digital health equity is a concept derived from health equity in the digital context (12), and it is a form of health equity. Its earliest core concept was the “digital divide,” which refers to disparities in the application and diffusion of digital technologies in healthcare. These disparities are reflected in older adults' unequal access to health information and healthcare services, as well as in the resulting differences in health status (4). Scholars such as Zhang and Tao (13) argue that, with the accelerating pace of digitalization, the digital divide, as a new form of social inequality, is exerting a profound influence on the allocation and utilization of health resources. The research results expand the theoretical research framework in the field of the digital divide and health inequality. In 2020, against the backdrop of the COVID-19 pandemic, Crawford and Serhal (14) proposed a digital health equity framework. This framework considers the various social, cultural, and economic factors that influence health and well-being, as well as their interactions. It is grounded in the process of social stratification within broader economic, cultural, and social contexts, in which age is one of the key factors shaping social stratification. The core contribution of this framework is its clear identification of how unequal access to technology may exacerbate the social gradient in health, underscoring the need for collaborative efforts to advance digital health equity at the individual, institutional, and societal levels. In 2022, Richardson et al. (15) proposed a framework for digital health equity, providing the first explicit definition of the concept as “everyone having a fair opportunity to participate in and benefit from digital health tools.” This framework also introduced a multilevel ecological analytical model that encompasses individual, interpersonal, community, and societal levels, moving beyond a singular resource perspective. Kaihlanen et al. (16), drawing on the digital health equity framework (DHEF) proposed by Crawford and Serhal, analyzed gaps in the determinants affecting vulnerable groups in the field of digital health and emphasized that older adults are among the vulnerable groups requiring particular attention. It is defined as ensuring that individuals have equal opportunities to benefit from the development and application of digital technology-related knowledge and practices to improve health. In 2023, Blanc et al. (17) refined Richardson's multi-level digital health equity ecosystem framework by integrating it with a model focused on a sense of belonging. This updated framework offers a more detailed analysis by categorizing barriers into four dimensions: individual, interpersonal, community, and institutional. It also connects these dimensions to specific digital interventions, such as digital health literacy and telemedicine. Furthermore, it defines digital health equity and inclusion as the “fair and just opportunities to use digital health tools to support positive health outcomes.” In 2024, Hatef et al. (18) proposed a digital health equity framework spanning the full lifecycle of digital health, including planning, development, procurement, implementation and maintenance, as well as monitoring, improvement, and equity assessment. Within this framework, older adults are identified as key stakeholders. By 2025, the concept of “digital health equity” is expected to gain significant attention, particularly concerning older adults. As society examines the challenges of an older population along with the rising prevalence of chronic diseases, it becomes clear that the topic of digital health equity for older adults requires urgent and in-depth exploration (19). Kokorelias et al. (20) utilized the PROGRESS-Plus framework to evaluate the equity of digital health interventions in managing heart failure among older adults. This study enhances our understanding of “digital health equity for older adults” by expanding it from a theoretical concept to tangible applications in disease management and the assessment of technology. Weng and Li (21) introduced the concept of “technological rights deprivation” among older adults from an ethical perspective. This concept broadens the understanding of digital health equity to include not only the ability to use technology but also whether individuals are acknowledged, included, and respected. As a result, it shifts the focus from merely technological intervention to a more compassionate approach to care. In the same year, Kim and Backonja (22) conducted a scoping review of frameworks and concepts related to digital health equity. The review concluded that digital health equity is a multilevel socio-ecological concept. Its goal is to ensure that everyone has fair and just opportunities to achieve the highest possible level of health through access to technology-supported health resources and services. In contrast to traditional theories of health equity, which focus on the distribution of physical medical resources and differences in regional and economic conditions, modern digital technologies are now an integral part of daily life and encompass a wide range of healthcare services. Additionally, digital transformation has become a leading trend in social development and the provision of public services. Building on the traditional connotation of health equity, digital health equity among older adults has evolved in response to changing times by incorporating the dimension of digital technology, thereby expanding the scope of research. The concept of equity has expanded from the fair allocation of physical medical resources to encompass the provision of digital resources, individual digital literacy, age-appropriate cultural adaptation, and comprehensive institutional safeguards throughout the entire process of digital technology use. As a result of the growing pressures from an older population and the high rates of chronic diseases, the concept of digital health equity for older adults has shifted. It has moved from merely “accessing technology” to “ensuring equal opportunities for participation and health outcomes within technology-driven chronic disease management systems.” This change represents the latest advancement in this area. Figure 2 illustrates the evolution of this concept.

### 3.2 Differentiating related terms

“Alternative terms” refer to words that have similar meanings to a certain concept and can be used interchangeably in specific contexts. A review of relevant literature at home and abroad indicates that words with meanings similar to “digital health equity” that can serve as alternative expressions mainly include “digital health justice” and “digital health fairness.”

Related terms are expressions that share conceptual similarities with a given concept, although they do not possess identical characteristics. Terms associated with digital health equity include “digital health equality”, “the digital health divide,” and “digital health literacy.” Digital health equality refers to providing the same digital health resources and opportunities to everyone. In contrast, digital health equity refers to ensuring that all individuals can achieve their optimal health outcomes through fair and inclusive access to digital health resources and opportunities (23). Digital health literacy is a key competency for achieving digital health equity. It refers to older adults' ability to seek, understand, evaluate, and apply electronic health information and services. Insufficient digital health literacy is a core mediating factor hindering the attainment of digital health equity, but it does not constitute equity itself (24). Although there is a broad consensus regarding the core definition of the digital health divide, scholars have framed it from different perspectives. In systematic reviews, it is generally defined as group-level disparities in access to and use of digital health resources (4). It is considered a key factor contributing to health inequalities among older adults and, in essence, a direct manifestation of the absence of digital health equity in this population (25). Although both digital health equity and the digital divide fall within the broader field of digital inequality research, they differ significantly in their theoretical positioning and analytical dimensions. From the perspective of the dynamic evolution of technology acquisition and use, the three-dimensional conceptual pyramid model frames the digital divide as comprising three progressive levels: access divide, usage divide, and domestication divide, reflecting a shift from access to use to proficient use (26). Digital health equity, grounded in the core value of health outcome equity, emphasizes that all individuals—regardless of age, gender, ethnicity, income, geographic location, or other social determinants—should have equal access to digital health services and comparable opportunities to achieve equitable health outcomes. It constitutes a multidimensional analytical framework encompassing social stratification, intermediary factors, digital health determinants, and health system components. Digital health equity goes beyond simply addressing hierarchical differences in individuals' ability to use digital technologies. Instead, it aims for the ethical goal of equality in health rights (27). This approach emphasizes the need to analyze unequal health outcomes and the social structural factors that contribute to them. As a result, the focus shifts from merely describing technological inequality to examining the governance surrounding health inequality.

The differences between these concepts—digital health equality, the digital health divide, digital health literacy, and digital health equity—are presented in Table 1.

**Table 1:** The differences among the concepts of digital health equity.

Type	Core focus	Goal orientation
Digital health equality	Equitable access to health resources and opportunities	Equal opportunity: involves ensuring equal access to digital health resources for all groups, thereby promoting non-discriminatory treatment from the start.
Digital health equity	Equitable health outcomes and social inclusion	Outcome equity: building on equal opportunity in resource access, this approach seeks to reduce health disparities associated with structural factors such as age, gender, income, and geography, enable all individuals to attain the highest possible standard of health, and uphold the ethical principle of equal health rights.
The digital health divide	Disparities among groups in access to and utilization of digital health resources	Identification and attribution of differences: the stratification of various groups regarding access to, use of, and effective engagement with digital health resources highlights deficiencies in digital health equity, which is a significant contributor to health inequality.
Digital health literacy	Capability to use digital health information and services	Intermediary empowerment: as a key capability for advancing digital health equity, an individual's ability to seek, understand, evaluate, and apply e-health information and services should be understood as a central mediating factor in the realization of equity, rather than as equity itself.

### 3.3 Determine the defining attributes

#### 3.3.1 Digital health resource equity

Digital health resource equity means providing older adults with equal and practical access to the necessary material resources and infrastructure to use digital health technologies, ensuring no discrimination. Infrastructure is a prerequisite for the operation of digital health technologies (28). Research indicates that disparities in older adults' access to technology significantly hinder their ability to independently acquire and effectively utilize health services (29). This disparity is primarily evident in urban-rural differences, socioeconomic status, and family resource disparities (29–31). Specifically, this is evident in the inadequate network coverage in rural areas, limited access to smart devices among economically disadvantaged older adults, and a lack of intergenerational support within families of older adults living alone.

#### 3.3.2 Digital access capability equity

Digital access capability equity in technology means individuals have fair opportunities at physical, cognitive, and value-identification levels to acquire, understand, and apply digital technologies and information in a digital health environment to maintain their health. Research has shown that older adults experiencing declines in visual, auditory, or motor functioning, along with lower educational attainment and cognitive impairment, often struggle to understand complex user interfaces, which significantly reduces their operational adaptability and efficiency in obtaining information (32, 33). Moreover, insufficient trust in digital technology substantially constrains their capacity to operate complex devices (21). In clinical practice, older adults with diabetes often encounter difficulties in completing the multi-step process of online registration because of age-related visual decline and reduced motor functioning. For some patients with mild cognitive impairment, barriers such as pop-up notifications and verification procedures may further discourage, or even prevent, their use of digital health services (34). Digital health literacy among older adults is a prerequisite for achieving digital health equity. Current research has shown that older adults require not only access to technology, but also the capacity to understand and critically use health information (35). Research by scholars such as Connolly et al. (36) suggests that older adults' active efforts to overcome learning barriers and usage-related burdens can effectively enhance the accessibility and user experience of digital health technologies.

#### 3.3.3 Digital cultural adaptation equity

Digital cultural adaptation equity refers to the equitable adaptation of digital health service systems to the needs of older adults through age-friendly service design, simplified operational processes, and optimized information presentation, to ensure that older adults can understand, accept, and use digital health products without barriers. Older adults have different health beliefs and behavioral habits compared to younger populations. In areas where traditional beliefs are still important, older adults tend to prefer verbal medical advice and face-to-face consultations. Therefore, digital health services should be designed to align with their cultural understanding and acceptance patterns (21). Many hospitals have introduced age-friendly service initiatives aimed at improving the experience for older adult patients. These initiatives include voice-enabled online registration, health education provided in local dialects, and in-person assistance at guidance desks. By simplifying operational procedures and catering to the communication preferences of older adults, these efforts have significantly increased acceptance and use of digital health services among older adult patients (37). Wilson et al. (38) found that when digital health technologies are designed to be user-friendly and culturally appropriate, they are more likely to promote equitable use among older adults. Table 2 presents the content descriptions of each attribute in the included studies.

**Table 2:** Description of the attributes of the included studies.

Categories	Description
**Digital health resource equity**	**Social resources:** Human (13, 44–47), Economic (13, 16, 20, 21, 31, 36, 46–54), Material resources (13, 14, 16, 20, 21, 25, 31, 34, 36, 39, 46, 48–50, 52, 54–58)
**Digital access capability equity**	**Functional:** Sociodemographic characteristics (19, 34, 37, 59), Factors related to physical and cognitive function (14, 16, 20, 21, 25, 36, 38, 41, 44, 48, 49, 55, 58, 60–63) **Social support network:** (16, 19, 31, 32, 37, 48, 49, 59, 61, 62, 64) **Negative attitudes:** Lack of trust in technology (14, 19, 20, 40, 41, 54, 58, 61, 64, 65), Privacy concerns (14, 32, 37, 39, 48, 50, 51, 59–61, 64), Hassle costs (38),Technophobia (56, 59) **Willingness to adopt digital empowerment:** Motivation to use (16, 19, 21, 34, 38, 51, 52, 56, 60, 62, 64, 66, 67), Self-efficacy (32, 37, 59, 61, 68), Autonomy (48, 65), Health-related beliefs (14, 37, 52), Digital literacy (13, 14, 16, 19, 21, 31, 32, 36, 39–41, 45, 47–49, 53, 54, 57–59, 61, 62, 64, 68–70)
**Digital cultural adaptation equity**	**Age-friendly renovation:** Visual presentation optimization (19, 21, 31, 37, 38, 40, 41, 46–49, 51, 55–57, 60–63, 66, 67, 71), User experience optimization (56), User-centered design (38, 39, 55, 60, 65, 66, 69, 71), Adaptation to diverse cultural needs (31), Alignment with values and preferences (16, 19, 34, 50, 62), Fairness in content and algorithms (48)

### 3.4 Antecedents of digital health equity among older adults

**Figure 3:**
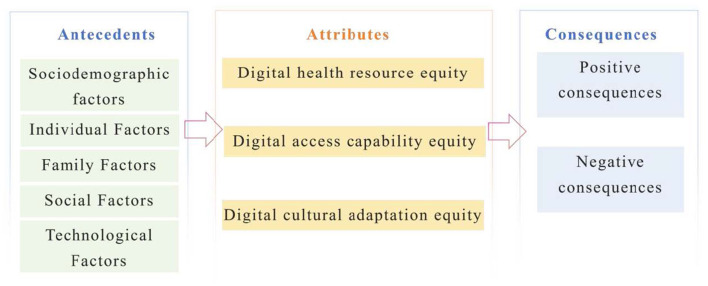
The antecedents, attributes, and consequences of digital health equity among older adults.

The antecedent variables of a concept generally refer to events or phenomena that precede its emergence (10). Many researchers have studied the concept of digital health equity. Through a thorough review of existing literature, several key factors influencing digital health equity among older adults have been identified. These factors include sociodemographic, individual, family, social, and technological factors (Figure 3).

#### 3.4.1 Sociodemographic factors

Research has shown that demographic characteristics—including age (39), gender (39), ethnicity (40), place of residence (29), educational attainment (33), income (33), and social status (30) constitute key variables affecting digital health equity among older adults. Older adults with lower educational attainment and limited health and digital literacy may struggle to use online patient service platforms (41, 42). Gallagher et al. (43) indicate that age is a significant determinant. Compared with their younger counterparts, the oldest-old are more likely to encounter difficulties in both owning mobile devices and using digital technologies.

#### 3.4.2 Individual factors

These factors span the physiological, cognitive, and psychological dimensions. Physiologically, older adults may experience blurred vision, diminished fine motor skills, and memory decline, which can significantly exacerbate disparities in digital competence within the older population (32). At the cognitive level, factors such as older adults' limited motivation for active learning and the rigidity of personal beliefs may hinder the pace at which they learn to use and adopt new products (48). At the psychological level, older adults' sense of alienation, insecurity, and distrust toward technology constitute substantial psychological barriers, thereby further reducing their willingness to adopt digital health services (72).

#### 3.4.3 Family factors

Support from family is crucial for promoting digital health equity among older adults (31). With the growing shift from traditional family structures to a “migratory-bird” family model, older adults are increasingly experiencing a lack of intergenerational digital support, which makes it difficult for younger family members to provide them with technical guidance (49). Xu et al. (73) found that individuals with higher household incomes were more likely to access and use telemedicine services.

#### 3.4.4 Social factors

Inadequate policy guidance, inequitable resource allocation, market supply bias, and insufficient social support are important factors affecting digital health equity among older adults. Yang et al. (74) reported that social relationships and social support are among the most influential factors affecting digital health equity. Older adults with support from their children or community volunteers are better able to overcome operational barriers, whereas socially isolated older adults are more likely to experience digital exclusion. Current health policy orientations have not adequately addressed the digital health needs of older adults. As a result, public resources have been allocated in a way that predominantly benefits younger and middle-aged populations. This has led to a significant bias in the delivery of digital health services, limiting older adults' access to equitable health care opportunities (48, 75).

#### 3.4.5 Technological factors

From a technical perspective, the lack of age-friendly design in digital health technologies constitutes a key factor influencing digital health equity among older adults (48). Research has shown that telemedicine and health monitoring devices are often characterized by operational complexity, cumbersome software interfaces, and design flaws such as misleading advertisements, which can create digital barriers at the point of use and thereby compromise digital health equity among older adults (55). Additionally, poorly performing health-related digital applications that lack rigorous empirical validation can lead to significant misjudgments regarding the health needs of older adults, thereby worsening inequalities in service distribution (76). Song et al. (59) found that older adults exhibited significantly greater technophobia regarding digital health than younger adults, particularly concerning privacy and security issues. Technophobia substantially constrains their willingness to adopt technologies such as video consultations and health monitoring applications.

### 3.5 Consequences of digital health equity among older adults

Consequences refer to the outcomes or resulting states that emerge from the inherent logic and intended objectives of a specific concept. The consequences of digital health equity among older adults include both positive and negative consequences (Figure 3).

#### 3.5.1 Positive consequences

The positive outcomes are mainly seen in better health results for older adults, the safeguarding of their health rights, and the alleviation of social burdens related to eldercare and healthcare. Fang et al (68) reported that the involvement of policymakers, technology developers, and healthcare providers in designing digital health technologies tailored to older adults can significantly enhance older adults' health self-efficacy, strengthen their initiative and confidence in health management, mitigate the adverse effects of the digital divide on their health rights, and ultimately improve their health outcomes. Through data-sharing mechanisms, family members can monitor the health status of older adults in real time, thereby reducing the need for frequent in-person visits and emergency interventions triggered by sudden health problems. These can significantly lessen the overall burden and stress of family caregiving, help divert demand away from traditional offline medical resources, and alleviate broader social burdens related to care for older adults and healthcare (77). Digital health infrastructure has been integrated into national strategies like rural revitalization. By ensuring fair distribution of digital health resources and optimizing services for older adults, it aims to reduce disparities in access to healthcare resulting from digital exclusion. This approach helps address digital health inequities among seniors and supports the achievement of healthy aging objectives in the digital era (48, 49).

#### 3.5.2 Negative consequences

Digital health inequity can have significant adverse effects on the physical and mental health of older adults through multiple pathways, including social interaction, psychological well-being, and health behaviors (33). If older adults are unable to adapt smoothly to the Internet era, this may not only weaken their sense of social participation and contribute to social isolation, but also reduce their social engagement and give rise to depressive symptoms (78). Cui (79) reported that the digital divide may generate a vicious cycle in the physical and mental health of older adults. Limited digital skills make it more difficult for older adults to obtain health information, thereby contributing to inadequate disease management and further diminishing their capacity to learn and use new technologies. At the same time, the pursuit of maximum efficiency in digital health services has led to the neglect of older adults' fundamental needs for dignity, emotional connection, and social participation, thereby producing a value deviation in technology empowerment (48).

### 3.6 Assessment tools

At present, there is no systematic assessment tool specifically designed to evaluate digital health equity among older adults. Although some studies have attempted to develop indicator systems for digital health equity (80), no standardized assessment instrument has been established. Moreover, these efforts have not sufficiently incorporated the physical and psychological characteristics of older adults or the actual contexts of digital technology use, making it difficult to comprehensively and accurately reflect the true level of digital health equity among older populations. Situational sensitivity emphasizes that assessment should encompass multiple scenarios, including home health monitoring, community health services, smart in-hospital diagnosis and treatment, and remote follow-up consultations and medication purchasing, while also considering individual differences in health status, caregiving environment, social support, and digital literacy (81). Future research needs to create specialized assessment tools that consider both age-friendliness and situational sensitivity. This approach will help identify equity gaps impacting older adults in digital health services and provide empirical evidence to guide the development of targeted intervention strategies, ultimately fostering a more inclusive digital health system.

### 3.7 Model cases of digital health equity among older adults

This study presents a case analysis of an older adult living at home to facilitate a better understanding of the concept and connotations of digital health equity among older adults (10). Mr. Wang, nearly 80 years old, is widowed, has a junior high school education, lives with diabetes, and resides in an older residential community. His daughter has worked away from home for many years, and he has long relied on regular check-ups at the local community health clinic. 1 day, the community introduced digital health management services for all residents, including remote monitoring, online consultations, and smart wearable devices. These services enable real-time monitoring of older adults' key vital signs, such as heart rate, blood pressure, and blood glucose. While using the wristband, Mr. Wang faced several challenges. After receiving the device, he discovered that the screen was too small and the font too fine, which made it difficult for him to measure his blood pressure and heart rate. Additionally, this model struggled to connect to the internet in his area, leading to delays in data transmission. In response to Mr. Wang's feedback, the designers optimized the wristband's interface, simplified the operating procedures, and enlarged the font, making it more culturally and age-appropriate (digital cultural adaptation equity). The community also equips older adults with free age-friendly smart health terminals, offers subsidized data plans, and establishes digital health service stations within the community service center (digital health resource equity). With the support of community volunteers, Mr. Wang was able to overcome both the psychological and technical barriers that had made him reluctant to use the device and hindered his understanding of how to operate it. After learning to use the health wristband, he showed a strong motivation to learn and became more proactive in using other digital health devices (digital access capability equity). After 1 year, the standardized management rate of hypertension among older adults in the community had increased by 30%, and the frequency of emergency visits had decreased significantly, thereby marking a substantive shift from technological inclusiveness to equity in health outcomes.

## 4 Discussion

### 4.1 Findings

Against the dual backdrop of deep population aging and the digital transformation of healthcare, digital health equity among older adults refers to a core social phenomenon concerning equality in older adults' access to, use of, and benefit from digital health services. Analyzing the concept of digital health equity among older adults allows healthcare providers to move beyond the surface issue of inadequate access to digital health services. It helps to identify the deeper equity concerns related to unequal resource allocation, insufficient technological adaptation, and a lack of social support. As a result, interventions can shift from a sole focus on digital skills training to comprehensive healthy services. These services should integrate resource optimization, technological adaptation, and a humanistic approach to care. At present, research on digital health equity among older adults in China remains at a preliminary exploratory stage. Future studies should further consider and explore the following aspects. First, there is a lack of locally developed assessment tools for evaluating digital health equity among older adults. Future research should establish a localized conceptual model and assessment system aligned with national conditions, grounded in the characteristics of China's older population and their digital use scenarios. It should also refine the multidimensional connotations of the concept and strengthen the age-friendliness and contextual sensitivity of the tools, so as to assess the level of digital health equity among older adults more accurately. Second, Intervention studies focused on digital health equity among older adults are still relatively limited in both China and internationally. Future research should build on the existing patterns of digital health usage among older adults in China, as well as the key challenges identified through conceptual analysis, including their causes and effects. This effort should aim to develop targeted, multilevel intervention programs. Additionally, it is important to refine policy design and service delivery systems related to digital health equity, enhance strategies for age-friendly technological adaptation and digital literacy, and provide practical evidence and policy guidance to support healthy aging.

### 4.2 Practical implications and clinical application

Clinical healthcare professionals can integrate a three-dimensional assessment framework for digital health equity among older adults into routine evaluations conducted during chronic disease follow-up and home-based care. Drawing on the three dimensions of equity—resources, capabilities, and cultural adaptability—they can accurately identify older adult patients' digital health deficits and develop stratified, individualized clinical intervention plans. For older adults in rural areas, low-income populations, and those living alone who face constraints in accessing resources, medical institutions can coordinate with primary healthcare systems to strengthen regional network infrastructure, expand access to smart devices, and mobilize intergenerational family support, thereby removing barriers to accessing digital healthcare hardware. For older adults whose physical and cognitive impairments limit their capacity to access and utilize resources effectively, interventions should prioritize training in practical device operation, digital health literacy, and the critical appraisal of health information. For older adults whose traditional healthcare beliefs make them less receptive to online diagnosis and treatment, efforts should focus on advancing age-friendly iterations of digital service platforms and optimizing service content to align with their established preferences for face-to-face medical consultations. The systematic implementation of three-dimensional intervention strategies can effectively narrow disparities in older adults' use of digital health services, improve the efficiency of primary-level health management for older populations and the inclusiveness of digital healthcare services, and enhance the quality and effectiveness of clinical services such as chronic disease management and home-based health monitoring.

## 5 Limitations

This study relies exclusively on publicly available literature for its theoretical analysis, without incorporating field research data on older adults in urban and rural settings or under different eldercare models. As a result, it lacks empirical support and does not sufficiently examine the practical manifestations or regional variations. At present, the academic community has yet to establish a unified theoretical framework for digital health equity among older adults, and few mature research frameworks are available for reference. This study failed to sufficiently differentiate the diversity within the older adult population concerning age, educational level, and disability status. Additionally, it does not provide a detailed discussion of the barriers that various groups encounter in accessing digital health resources, along with their corresponding rights and interests. Future research could integrate qualitative interviews with large-sample surveys to further refine conceptual definitions and develop a standardized quantitative evaluation system.

## 6 Conclusion

This study uses Rodgers' evolutionary concept analysis method to explore the defining attributes, antecedents, consequences, and related concepts of digital health equity among older adults. It also provides clearer case examples to enhance understanding of its meaning. A clear definition of concepts provides researchers with a theoretical framework to support healthcare professionals in developing digital health equity scales for older adults, designing personalized interventions, and conducting quantitative research. Simultaneously, this framework can serve as a reference for aging and public health management departments to optimize supportive public policies, improve inclusive digital health services, facilitate the standardized management of chronic diseases among older adults, and promote the orderly implementation of healthy aging strategies across multiple dimensions. This study's analysis of the concept of digital health equity among older adults reflects only the current state of research. Digital health equity among older adults is a dynamic, multidimensional issue of social justice, and the meaning of this concept still requires further exploration and refinement.

## Data Availability

The original contributions presented in the study are included in the article/Supplementary material, further inquiries can be directed to the corresponding author.

## References

[1] Ebo TO Clement David-Olawade A Ebo DM Egbon E Olawade DB. Transforming healthcare delivery: a comprehensive review of digital integration, challenges, and best practices in integrated care systems. Digit Eng. (2025) 6:100056. doi: 10.1016/j.dte.2025.100056

[2] Yuguero O Ruiz-Trujillo P Esquerda M Terribas N Aymerich M. Applying the principle of justice in digital health. Npj Digit Med. (2025) 8. doi: 10.1038/s41746-025-01877-8 PMC12280129 40691313

[3] Pong C Tseng R Tham YC Lum E. Current Implementation of digital health in chronic disease management: scoping review. J Med Internet Res. (2024) 26:e53576. doi: 10.2196/53576 39666972 PMC11671791

[4] Yang YB Ma CY. A. review of research on the digital health divide at home and abroad. Libr Inf Sci. (2024) 41:154–66. doi: 10.13366/j.dik.2024.03.154

[5] Zheng X Zhou J Hu Y Xu M Chen MJ Gong HP . A qualitative study on the digital health technology use experience of elderly cancer patients. J Nurs. (2023) 38:20-4+34.

[6] Hepburn J Williams L McCann L. Barriers to and facilitators of digital health technology adoption among older adults with chronic diseases: updated systematic review. JMIR Aging. (2025) 8:e80000. doi: 10.2196/80000 40934502 PMC12464506

[7] Han RX Fu JJ Liu SQ Lu Y. Research progress on the application of “Internet” medical services in the older population. Nurs Res. (2021) 35:3657–60. Available online at: https://kns-cnki-net-443.webvpn.ybu.edu.cn/kcms2/article/abstract?v=H26UkN0bSyR2ucPO4fUCc47pCAANpOolJBG8_osQm1jGOcLmF8F4M9PbsstbEKZjAGbUE3Ye9wcr7G-BS6NueS0MNAOyeODVkCjbtjwqI5vJue6DTOaNkOUghU3xVCcsXPYDInFW6nQwv9UNrPAh0ZBf9GWB91qwYCYRp_xE7g33B6ycPSb9uQ==&uniplatform=NZKPT&language=CHS

[8] Shi Y Ma D Zhang J Chen B. In the digital age: a systematic literature review of the e-health literacy and influencing factors among Chinese older adults. Z Gesundh Wiss. (2023) 31:679–87. doi: 10.1007/s10389-021-01604-z 34104627 PMC8175232

[9] Dong JK Qian H Xing YQ Zhang DL Wang HM. Digital health equity: theoretical perspectives and influencing factors. Health Econ Res. (2024) 41:5–9.

[10] Rodgers BL Jacelon CS Knafl KA. Concept analysis and the advance of nursing knowledge: state of the science. J Nurs Scholarsh. (2018) 50:451–9. doi: 10.1111/jnu.12386 29689127

[11] Li GQ Hu JL Zheng YW Ruan H. Concept analysis methods and their application in nursing research. J Nurs. (2018) 33:100−2. Available online at: https://kns-cnki-net-443.webvpn.ybu.edu.cn/kcms2/article/abstract?v=H26UkN0bSyS6YJmL6O1L0ZZmnMK51hXpJLN_YMO-nvroMywmK4HDwogG43iU4mB_0FC5YZUGpj7qri2rtX0wcKQ7Xoa0l-gZdgEoOj9PtNtu2PlomIQlAykzBqEOho89yE2rqkxR0VA7cgnIfwdI9mlLke8bQ5by2IbjrZ31bDX7nRQy5vmhmA = =&uniplatform=NZKPT&language=CHS

[12] Lyles CR Nguyen OK Khoong EC Aguilera A Sarkar U. Multilevel determinants of digital health equity: a literature synthesis to advance the field. Annu Rev Public Health. (2023) 44:383–405. doi: 10.1146/annurev-publhealth-071521-023913 36525960 PMC10329412

[13] Zhang SJ Tao JK. Digital divide, social capital, and health inequality. Northwest popul. (2026) 47:14–28. doi: 10.15884/j.cnki.issn.1007-0672.2026.03.002

[14] Crawford A Serhal E. Digital Health Equity and COVID-19: The innovation curve cannot reinforce the social gradient of health. J Med Internet Res. (2020) 22:e19361. doi: 10.2196/19361 32452816 PMC7268667

[15] Richardson S Lawrence K Schoenthaler AM Mann D. A framework for digital health equity. Npj Digit Med. (2022) 5:6. doi: 10.1038/s41746-022-00663-0 35982146 PMC9387425

[16] Kaihlanen AM Virtanen L Buchert U Safarov N Valkonen P Hietapakka L . Towards digital health equity-a qualitative study of the challenges experienced by vulnerable groups in using digital health services in the COVID-19 era. BMC Health Serv Res. (2022) 22:188 doi: 10.1186/s12913-022-07584-4 35151302 PMC8840681

[17] Blanc J Hahn K Oliveira B Phillips R Duthely LM Francois L . Bringing health care equity to diverse and underserved populations in sleep medicine and research through a digital health equity framework. Sleep Med Clin. (2023) 18:255–67. doi: 10.1016/j.jsmc.2023.05.009 37532367 PMC10300114

[18] Hatef E Hudson Scholle S Buckley B Weiner JP Austin JM. Development of an evidence- and consensus-based digital healthcare equity framework. JAMIA Open. (2024) 7:ooae136. doi: 10.1093/jamiaopen/ooae136 39553827 PMC11565864

[19] Li C Wang H Yuan J Shi L Chen Y Gao Z . Current status of older people with chronic diseases adopting digital health technologies: a scoping review. Digit Health. (2025) 11:20552076251348578. doi: 10.1177/20552076251348578 40487886 PMC12144359

[20] Kokorelias KM Hoang P Harris MT. Who gets included? A scoping review protocol of digital health interventions for older adults with heart failure through an equity lens. PLoS ONE. (2025) 20:e0337990. doi: 10.1371/journal.pone.0337990 41343488 PMC12677474

[21] Weng LC Li L. Health justice and the right to technology: an ethical analysis of equity and accessibility issues in digital healthcare practices for older adults. Sci Soc. (2025) 15:133–49. doi: 10.19524/j.cnki.10-1009/g3.2025.01.133

[22] Kim KK Backonja U. Digital health equity frameworks and key concepts: a scoping review. J Am Med Inform Assoc. (2025) 32:932–44. doi: 10.1093/jamia/ocaf017 39936843 PMC12012343

[23] de Souza RR. Health policy and practice towards equity. Rev Esc Enferm Usp. (2007) 41:765–70. doi: 10.1590/S0080-62342007000500004 20608374

[24] Norman CD Skinner HA. eHealth Literacy: Essential Skills for Consumer Health in a Networked World. J Med Internet Res. (2006) 8:e9. doi: 10.2196/jmir.8.2.e9 16867972 PMC1550701

[25] Wu M Xue Y Ma C. The association between the digital divide and health inequalities among older adults in china: nationally representative cross-sectional survey. J Med Internet Res. (2025) 27:e62645. doi: 10.2196/62645 39813666 PMC11780301

[26] Wei L. The Digital Divide: Concepts, Causes, and Consequences. Hangzhou: Zhejiang University Press. (2024).

[27] Razaq S Allana S. Understanding digital health equity: a conceptual analysis. J Adv Nurs. (2025). doi: 10.1111/jan.70405 41312781 PMC13356374

[28] Labrique A. From infrastructure to impact: why foundations matter in digital health. Bull World Health Organ. (2025) 103:83-a. doi: 10.2471/BLT.24.293085 39897637 PMC11774216

[29] D'Amico R Schnell PM Foraker R Olayiwola JN Jonas DE Bose S. The evolution of primary care telehealth disparities during COVID-19: retrospective cohort study. J Med Internet Res. (2023) 25:e43965. doi: 10.2196/43965 37146176 PMC10233430

[30] Wang FQ. The health digital divide: how is the internet changing health inequality? Sociol Rev. (2025) 13:5–28. Available online at: https://kns-cnki-net-443.webvpn.ybu.edu.cn/kcms2/article/abstract?v=H26UkN0bSyQ6ceIKo8SPaGLctnWF71ck8eiJXbGvZW0OHc9k_RgDpSCVFyRbeUS3irso0hFofeiMes5hfqLSs7mp2kkX93D4AvkCrs9RDpZdImuaU31slB5A1j7P3RgyQHOWy3dJWHvL_JQL_uaIZUAi3KyxMTPRiWv37vea6g6447VfWMp6Fg==&uniplatform=NZKPT&language=CHS

[31] Veras M Auger LP Sigouin J Gheidari N Nelson MLA Miller WC . Ethics and equity challenges in telerehabilitation for older adults: rapid review. Jmir Aging. (2025) 8. doi: 10.2196/69660 40802966 PMC12349735

[32] Hirvonen N Enwald H Känsäkoski H Eriksson-Backa K Nguyen H Huhta AM . Older adults' views on eHealth services: a systematic review of scientific journal articles. Int J Med Inform. (2020) 135:104031. doi: 10.1016/j.ijmedinf.2019.104031 31918340

[33] Tian YH Mu FJ. The impact of the digital divide on older adult: an empirical analysis based on CGSS 2021. Sci Econ soc. (2025) 43:56–74. doi: 10.19946/j.issn.1006-2815.2025.02.006

[34] Wang YR Wang Q Li LN Wang K Liu SQ He Q . Survey of diabetic patients' willingness to use mobile medical services and its influencing factors. Gen Pract Chin. (2017) 20:1619−25. Available online at: https://kns-cnki-net-443.webvpn.ybu.edu.cn/kcms2/article/abstract?v=H26UkN0bSyQEW4I9D6-P3sADNKgZqFv2hPcIL_P6YVmDrRSZg9h3dFQ4289bLSF-DZ3I8EuEDcJCOu_2g56gYv4CxJNyvudoOIDbN7hdAsRppelcsOWll01aaQnAwOFqsA6iUtt3C9E7l3pWqueRjgQeW-erTwdwzIwbLG2IFgpzBm8zqZQnHg==&uniplatform=NZKPT&language=CHS

[35] Cao GH Dong HQ Chen YF. Research on the construction of a digital health literacy framework and cultivation path for older adults under the context of smart elderly care. J Inf Sci. (2025) 44:1031–44. Available online at: https://kns-cnki-net-443.webvpn.ybu.edu.cn/kcms2/article/abstract?v=H26UkN0bSyTcIgFkGfWNJsHczQTcdkMyCW-Pufw4A_-XNOQNgTCL4zMsuwcLX007feuIRZOsz2a6Dc8UhzMMCAd9JU4gW_T9s0gxUV9KyfFshVpSLjDMMDKLAYVRDCgDj2Tg66l8P9JgckOeNs1zbCU-MLjI07C7_1BukJAv2cnfH5BppuwjSQ==&uniplatform=NZKPT&language=CHS

[36] Connolly G Costa-Font J Srivastava D. Did COVID-19 reduce the digital divide? A systematic review. Health Policy Technol. (2025) 14:100979. doi: 10.1016/j.hlpt.2025.100979

[37] Zhang Q Ke HB. Rural older adult patients' willingness to use telemedicine from the perspective of emotional atmosphere: a qualitative study in S village, L county, Z province. J Party Sch CPC Hangzhou Munic Comm. (2024) 3:84–96.

[38] Wilson S Tolley C Mc Ardle R Lawson L Beswick E Hassan N . Recommendations to advance digital health equity: a systematic review of qualitative studies. NPJ Digit Med. (2024) 7:173. doi: 10.1038/s41746-024-01177-7 38951666 PMC11217442

[39] Yang S Cha MJ van Kessel R Warrier G Thrul J Lee M . Understanding inequalities in mobile health utilization across phases: systematic review and meta-analysis. Int J Med Inform. (2025) 27:e71349. doi: 10.2196/71349 40811740 PMC12352709

[40] Budhwani S Fujioka J Thomas-Jacques T De Vera K Challa P De Silva R . Challenges and strategies for promoting health equity in virtual care: findings and policy directions from a scoping review of reviews. J Am Med Inform Assoc. (2022) 29:990–9. doi: 10.1093/jamia/ocac022 35187571 PMC9006706

[41] Roy S Lartey ST Durneva P Jha N Ofori MA Zeba Z . Digital health technology infrastructure challenges to support health equity in the United States: scoping review. J Med Internet Res. (2025) 27:e70856. doi: 10.2196/preprints.70856 40958727 PMC12441647

[42] Weiss D Rydland HT Øversveen E Jensen MR Solhaug S Krokstad S. Innovative technologies and social inequalities in health: a scoping review of the literature. PLoS ONE. (2018) 13:e0195447. doi: 10.1371/journal.pone.0195447 29614114 PMC5882163

[43] Gallagher R Roach K Sadler L Glinatsis H Belshaw J Kirkness A . Mobile technology use across age groups in patients eligible for cardiac rehabilitation: survey study. JMIR Mhealth Uhealth. (2017) 5:e161. doi: 10.2196/mhealth.8352 29066425 PMC5676027

[44] Cui Y He Y Xu X Zhou L Nutakor JA. The impact of digital social capital on the health of older adults: a moderated mediation effect test. Digit Health. (2024) 10:20552076241253095. doi: 10.1177/20552076241253095 40290729 PMC12032472

[45] Nouri S Khoong Elaine C Lyles Courtney R Karliner L. Addressing equity in telemedicine for chronic disease management during the Covid-19 pandemic. Catal Non-issue Content. (2020) 1. doi: 10.1056/CAT.20.0123

[46] Batsis JA DiMilia PR Seo LM Fortuna KL Kennedy MA Blunt HB . Effectiveness of ambulatory telemedicine care in older adults: a systematic review. J Am Geriatr Soc. (2019) 67:1737–49. doi: 10.1111/jgs.15959 31066916 PMC6684409

[47] Gordon NP Hornbrook MC. Differences in access to and preferences for using patient portals and other eHealth technologies based on race, ethnicity, and age: a database and survey study of seniors in a large health plan. J Med Internet Res. (2016) 18:e50. doi: 10.2196/jmir.5105 26944212 PMC4799429

[48] Song QC Zhang Q. Challenges and path choices for healthy aging in the digital era. J Jinan Univ. (2025) 35:16–27. doi: 10.20004/j.cnki.ujn.2025.04.002

[49] Jiang YJ Shao YZ Zhang HJ Zhou CY. Digital dilemma and breakthroughs of left-behind older adults in rural Nanjing under the background of digital health. J Nanjing Med Univ. (2024) 24:343–51.

[50] Fang ML Korol A Sixsmith J Siden E Demestihas M-A Sixsmith A. A scoping review exploration of the intended and unintended consequences of eHealth on older people: a health equity impact assessment. Hum Technol. (2018) 14:297–323. doi: 10.17011/ht/urn.201811224835

[51] Luo J Zhang K Huang Q Jiang S Pan Y. From acceptance to dependence: exploring influences of smart healthcare on continuous use intention of mobile health services among older adults with chronic illnesses in China. Behav Sci. (2024) 15:19. doi: 10.3390/bs15010019 39851823 PMC11762675

[52] Jaana M Tamim H Paré G. eHealth and the digital divide among older canadians: insights from a national cross-sectional study. J Med Internet Res. (2025) 27:e72274. doi: 10.2196/72274 41289575 PMC12646552

[53] Lee J Jang SN. Have changes in Internet use during the COVID-19 pandemic affected older adults? self-rated health? A cross-sectional study of young-old and old-old populations in Korea. Br Geriatr Nurs. (2022) 48:1–5. doi: 10.1016/j.gerinurse.2022.09.012 36219933 PMC9510094

[54] Man REK Ho AXY Lee EPX Fenwick EKD Aravindhan A Ho KC . Awareness and attitudes of elderly Southeast Asian adults towards telehealth during the COVID-19 pandemic: a qualitative study. Singapore Med J. (2025) 66:256–64. doi: 10.4103/singaporemedj.SMJ-2022-117 37675683 PMC12161648

[55] Dong JK Wang HM Xing YQ Zhang DL. Value co-creation and technological innovation: how to resolve equity issues in the digital transformation of health and hygiene—an analysis based on the case of age-friendly adaptation of medical insurance codes. E-government. (2025):87–99.

[56] Portz JD Bayliss EA Bull S Boxer RS Bekelman DB Gleason K . Using the technology acceptance model to explore user experience, intent to use, and use behavior of a patient portal among older adults with multiple chronic conditions: descriptive qualitative study. J Med Internet Res. (2019) 21:e11604. doi: 10.2196/11604 30958272 PMC6475817

[57] Kampmeijer R Pavlova M Tambor M Golinowska S Groot W. The use of e-health and m-health tools in health promotion and primary prevention among older adults: a systematic literature review. BMC Health Serv Res. (2016) 16 Suppl 5:290. doi: 10.1186/s12913-016-1522-3 27608677 PMC5016733

[58] Christensen LF Moller AM Hansen JP Nielsen CT Gildberg FA. Patients' and providers' experiences with video consultations used in the treatment of older patients with unipolar depression: a systematic review. J Psychiatr Ment Health Nurs. (2020) 27:258–71. doi: 10.1111/jpm.12574 31677331

[59] Song X Li S Dong L Li Y Yang X Bao J . Technophobia in digital health contexts: a systematic review and meta-analysis with a focus on older adults. Digit Health. (2025) 11:20552076251374218. doi: 10.1177/20552076251374218 40918073 PMC12409025

[60] Sinabell I Ammenwerth E. Challenges and recommendations for eHealth usability evaluation with elderly users: systematic review and case study. Univers Access Inf Soc. (2024) 23:455–74. doi: 10.1007/s10209-022-00949-w

[61] Zhao YC Zhao M Song S. Online health information seeking behaviors among older adults: systematic scoping review. J Med Internet Res. (2022) 24:e34790. doi: 10.2196/34790 35171099 PMC8892316

[62] Jiang Y Sun P Chen Z Guo J Wang S Liu F . Patients' and healthcare providers' perceptions and experiences of telehealth use and online health information use in chronic disease management for older patients with chronic obstructive pulmonary disease: a qualitative study. BMC Geriatr. (2022) 22:9. doi: 10.1186/s12877-021-02702-z 34979967 PMC8721473

[63] Liu N Yin J Tan SS Ngiam KY Teo HH. Mobile health applications for older adults: a systematic review of interface and persuasive feature design. J Am Med Inform Assoc. (2021) 28:2483–501. doi: 10.1093/jamia/ocab151 34472601 PMC8510293

[64] Tanaka M Ishii S Matsuoka A Tanabe S Matsunaga S Rahmani A . Perspectives of Japanese elders and their healthcare providers on use of wearable technology to monitor their health at home: a qualitative exploration. Int J Nurs Stud. (2024) 152:104691. doi: 10.1016/j.ijnurstu.2024.104691 38262231

[65] Karlsen C Ludvigsen MS Moe CE Haraldstad K Thygesen E. Experiences of community-dwelling older adults with the use of telecare in home care services: a qualitative systematic review. JBI Database Syst Rev Implement Rep. (2017) 15:2913–80. doi: 10.11124/JBISRIR-2017-003345 29219874

[66] Arsenijevic J Tummers L Bosma N. Adherence to electronic health tools among vulnerable groups: systematic literature review and meta-analysis. J Med Internet Res. (2020) 22:e11613. doi: 10.2196/11613 32027311 PMC7055852

[67] Foster M Xiong W Quintiliani L Hartmann CW Gaehde S. Preferences of older adult veterans with heart failure for engaging with mobile health technology to support self-care: qualitative interview study among patients with heart failure and content analysis. JMIR Form Res. (2022) 6:e41317. doi: 10.2196/41317 36538348 PMC9812271

[68] Fang Z Liu Y Peng B. Empowering older adults: bridging the digital divide in online health information seeking. Humanit Soc Sci Commun. (2024) 11:1748. doi: 10.1057/s41599-024-04312-7

[69] Matthew-Maich N Harris L Ploeg J Markle-Reid M Valaitis R Ibrahim S . Designing, implementing, and evaluating mobile health technologies for managing chronic conditions in older adults: a scoping review. JMIR Mhealth Uhealth. (2016) 4:e29. doi: 10.2196/mhealth.5127 27282195 PMC4919548

[70] Smith SG O'Conor R Aitken W Curtis LM Wolf MS Goel MS. Disparities in registration and use of an online patient portal among older adults: findings from the LitCog cohort. J Am Med Inform Assoc. (2015) 22:888–95. doi: 10.1093/jamia/ocv025 25914099 PMC4810779

[71] Vergouw JW Smits-Pelser H Kars MC van Houwelingen T van Os-Medendorp H Kort H . Needs, barriers and facilitators of older adults towards eHealth in general practice: a qualitative study. Prim Health Care Res Dev. (2020) 21:e54. doi: 10.1017/S1463423620000547 33263272 PMC7737186

[72] Arcury TA Sandberg JC Melius KP Quandt SA Leng X Latulipe C . Older adult internet use and eHealth literacy. J Appl Gerontol. (2020) 39:141–50. doi: 10.1177/0733464818807468 30353776 PMC6698430

[73] Xu Z Zhou L Gu W Yang Z Zhang L. Telehealth, medical decisions and new health inequality in China. BMC Public Health. (2025) 25:793. doi: 10.1186/s12889-025-22039-1 40011865 PMC11866627

[74] Yang YB Ma CY Liu HP Lv SY Liao WZ. Based on capital theory to exploring the digital health divide and determinants among urban and rural older adults in China: Cross-sectional study. Digit Health. (2025) 11. doi: 10.1177/20552076251374122

[75] Zhang JP Cheng MW Gong XM. Research on the characteristics and influencing factors of the digital divide between urban and rural areas in China. Stat Inf Forum. (2021) 36:92–102.

[76] Wang WT Lin X Wu Q Dong TT. Governance challenges and optimization strategies for digital health literacy among older adults: based on actor-network theory. Health Econ Res. (2025) 42:16−9.

[77] Cui Y Bao H Wen K Wen H. Bridging the digital health divide: digital endowment, informal social participation, and health inequality among older adults. BMC Health Serv Res. (2025) 26:99. doi: 10.1186/s12913-025-13872-6 41408271 PMC12822261

[78] Kawashima M Uchino M Kawazoe T Kamiyashiki M Sano K Tsubota K . Field test of web-based screening for dry eye disease to enhance awareness of eye problems among general internet users: a latent strategy to promote health. J Med Internet Res. (2013) 15:102–11. doi: 10.2196/jmir.2198 24072379 PMC3785952

[79] Cui A. The silver digital divide: challenges of internet technology to the physical and mental health of the elderly. E-commer Rev. (2024) 13. doi: 10.12677/ecl.2024.132413

[80] Maaß L Badino M Iyamu I Holl F. Assessing the digital advancement of public health systems using indicators published in gray literature: narrative review. JMIR Public Health Surveill. (2024) 10:e63031. doi: 10.2196/63031 39566910 PMC11618018

[81] McKenna VB Mathew JB Finn Y Sixsmith J. Approaches to assessment of community level health literacy: a scoping review. Health Promot Int. (2025) 40. doi: 10.1093/heapro/daaf123 41124601 PMC12543020

