# Supplementary material for: Digital health equity among older adult(s): a conceptual analysis
Source: Front Public Health. 2026 Jul 6;14:1891578. doi: 10.3389/fpubh.2026.1891578 (PMC13381788; doi:10.3389/fpubh.2026.1891578)
Supplement: Supplementary file 1 [file Table_1.DOCX]

Table1 Characteristics of the studies included.

| Article | Year | Study country | Design | Attributes | Antecedents | Consequences |
| --- | --- | --- | --- | --- | --- | --- |
|  |  |  |  | **·Digital health resource equity**  **·Digital access capability equity**  **·Digital cultural adaptation equity** | **·Sociodemographic factors**  **·Individual factors**  **·Family factors**  **·Social factors**  **·Technological factors** | ·**Positive consequences**  **·Negative consequences** |
| Zhang SJ et al.(13) | 2025 | China | Quantitative | Inadequate infrastructure, limited financial capacity, insufficient digital social capital, poor discernment, limited motivation to explore, and low willingness to use digital technologies. | Inequalities in the digitalization process across gender, age, and urban–rural.  Family ties primarily help digitally disadvantaged groups enhance access to information and improve health-related decision-making through a “digital feedback” mechanism.  The digital divide exacerbates health inequalities by weakening social networks. | Digital support alleviates the difficulties faced by older adults.  Social isolation and social participation. |
| Crawford A et al.(14) | 2020 | Canada | Conceptual framework | Lack of infrastructure,  Limited ability to evaluate information,  Insufficient motivation to explore,  Limited motivation to use digital technology,  Physical and cognitive limitations,  Lack of trust in technology | An individual’s social position shapes health risks and vulnerability.  Factors such as unemployment, poverty, educational attainment, and prior exposure to digital media can all influence access to digital health resources.  Tendencies to avoid seeking medical care or to downplay health risks may further contribute to resistance to digital health services. Privacy concerns also play an important role.  Limitations in the healthcare system and insufficient community resources may further constrain access.  Users may lack the ability to assess the quality of digital health information. | Improve health outcomes.  Alleviating digital health inequalities. |
| Kaihlanen AM et al.(16) | 2022 | Finland | A qualitative  study | Lack of basic computer skills and access to Appropriate devices.  distrust in the quality of telemedicine services and Declining memory capacity.  reliance on in-person medical visits and fixed Health-seeking attitudes.  Financial difficulties.  Poor website usability and complex operational procedures.  Concerns about personal data privacy breaches. | Financial constraints.  Lack of compatible devices.  Poor physical health.  Complicated operation and an unfriendly interface.  Privacy and security concerns that hinder use. | Digital services lack a personal touch, nonverbal communication, and emotional interaction, resulting in a sense of communicative distance and an inability to meet interpersonal emotional needs. |
| Li C et al.(19) | 2025 | China | A scoping Review | Support from family care, community volunteers, and medical institutions between generations.  Lacking elder-friendly design, complicated to operate, and the interface is annoying. | lack of interest, resistance to new technology, and low motivation to learn.  Privacy concerns.  Lack of age-friendly design, complicated operations, and cumbersome interfaces.  Insufficient social support. | Enhance older adults’ self-efficacy and initiative in using digital technologies, thereby improving their health and overall quality of life. |
| Kokorelias KM et al.(20) | 2025 | Canada | A scoping Review | Lack of infrastructure.  Physical and cognitive impairments. | Older adults often face difficulties in using digital health services.  Poverty, racial identity, geographic remoteness, language barriers, and limited digital literacy constitute major obstacles to their use of online healthcare services.  Socially isolated older adults are also more vulnerable to digital exclusion.  Poor usability and high operational barriers further constrain access. | NR |
| Weng LC et al.(21) | 2025 | China | Qualitative | Insufficient economic resources and lack of basic infrastructure.  Low levels of digital literacy and physical and cognitive impairments.  Most digital health products lack age-friendly design. | Physical decline and memory deterioration.  Concerns about privacy breaches and skepticism toward online healthcare. | Digital technology is driven by efficiency and profit, often neglecting the needs of older adults and losing its human-centered character.  This creates a vicious cycle of low digital literacy, difficulty in evaluating information, and lagging learning capacity. |
| Wu M et al.(25) | 2025 | China | Quantitative | Urban–rural disparities.  Intergenerational family support.  Physical and cognitive impairments. | Lack of age-friendly design and operational complexity.  Unequal allocation of public resources.  Insufficient social support.  Decline in physical and cognitive functioning. | Expanding urban and rural network coverage and promoting a more equitable distribution of resources can help digital inclusion reduce health inequalities among older adults. |
| Veras M et al.(31) | 2025 | Canada | Review | Urban–rural disparities and infrastructural shortcomings.  Physical and cognitive impairments.  User-friendly design. | Visual and sensory impairments, as well as declines in fine motor skills.  Low willingness to learn.  Insufficient family support.  Privacy concerns that hinder use. | Multiple barriers, including limited digital literacy and operational difficulties, hinder use and gradually weaken both the willingness and the ability to learn digital technologies, thereby creating a vicious cycle. |
| Hirvonen N et al.(32) | 2020 | Finland | A systematic review | Limited prior experience. Concern about data loss or device misplacement. Dependence on professional assistance. | Unfamiliarity.  Inadequate infrastructure .  Issues related to availability and ease of use. | Changes in actual health behaviors.  Anxiety, stress, isolation, and reduced social interaction. |
| WangYR et al.(34) | 2017 | China | Quantitative | Education level influences both the ability and willingness to use the technology.  Privacy leakage is a common concern among patients, and perceived risk can negatively affect their willingness to use the technology.  The technology may be perceived as difficult to operate. | Middle-aged and older adults tend to adopt new mobile healthcare technologies more slowly and may show lower levels of acceptance.  Patients may perceive risks related to privacy and security.  The attitudes of family members and friends can influence willingness to use the technology.  If the product is difficult to operate and not easy to learn, willingness to use it may decrease.  Positive attitudes from family members and healthcare professionals can increase willingness to use the technology. | Older adults and people with lower levels of education often have limited digital skills and understanding, which can reduce their willingness to use technology and make them more resistant to learning new skills. |
| Connolly G et al.(36) | 2025 | UK | Review | People facing financial difficulties may lack access to devices or the internet.  Physical or cognitive impairments can make digital tools difficult to use.  Limited digital literacy can create barriers to use.  Concerns about the reliability or safety of digital technology may reduce trust. | Older adults may find it difficult to use.  Differences in gender, place of residence, education level, income, digital literacy, and social status can all affect use.  Some people may be unwilling to learn or may hold fixed mindsets.  Sensory functions may decline with age.  Feelings of insecurity and distrust may discourage use.  The operation process may be too complicated. | People facing financial difficulties may not have access to devices or the internet. Physical or cognitive challenges can make digital tools difficult to use. Limited digital literacy can also create barriers. Concerns about the safety and reliability of digital technology may further reduce trust. |
| ZhangQ et al.(37) | 2024 | China | Qualitative | A lack of trust in digital technology can hinder acceptance.  Some individuals may feel that they lack sufficient knowledge or experience, which makes telemedicine difficult to understand and accept.  Deep-rooted traditional attitudes toward seeking medical care may also reduce acceptance of telemedicine. | Older adults of different ages, living areas, education levels, and levels of digital literacy may face difficulties in using digital healthcare services.  Many older adults continue to rely on traditional in-person medical care.  High-quality medical resources have not been sufficiently extended to rural areas.  The system’s operating procedures may be difficult to understand. | Older adults in rural areas often have limited digital and cognitive abilities, which can make it difficult for them to use telemedicine services or access health information. As a result, they may become more resistant to learning new technologies. |
| Wilson S et al.(38) | 2024 | UK | A systematic review | Visual and hearing impairments may make digital health technologies more difficult to use.  Operational difficulties and other usage barriers can reduce accessibility.  A lack of trust in technology may discourage use.  User-friendly, age-friendly design can increase adoption among the target population. | Differences in gender, ethnicity, place of residence, education level, income, social status, and digital literacy can influence access and use.  Visual or other sensory impairments may create barriers to use.  A lack of trust can reduce willingness to adopt the technology.  An imbalanced allocation of public resources may limit access to services.  A misunderstanding of older adults’ needs in the design process may reduce usability and acceptance. | Infrastructure development and inclusive design can help reduce inequalities in digital health.  Digital services tend to prioritize efficiency while overlooking the needs of older adults. |
| Yang S et al.(39) | 2025 | Korea | Systematic review and meta-analysis | Social and economic status can affect access to and use of resources.  Age, physical condition, and overall health may create barriers to use.  Previous experience with technology, operational complexity, and trust-related concerns can also influence use. | Older adults often face difficulties in owning mobile devices and using digital technologies.  A lack of social support can contribute to digital exclusion.  Most mobile health applications have poor usability and are complicated to operate. | A lack of digital skills can limit their ability to use mobile health services and access health information. |
| Budhwani S et al.(40) | 2022 | Canada | A scoping Review | A lack of digital literacy and practical skills can hinder use.  Trust issues related to digital technology may reduce willingness to adopt it.  Traditional attitudes toward medical treatment may also act as a barrier. | A lack of policy guidance and imbalanced resource allocation can hinder the development and use of telemedicine.  Feelings of insecurity, distrust, and concerns about privacy may reduce willingness to use telemedicine.  A lack of family support can also hinder the use of telemedicine. | There is a mismatch between the intended values of technology and its practical application. |
| Roy S et al.(41) | 2025 | USA | A scoping Review | Physical and cognitive impairments.  Trust and value alignment.  Product design and cultural. | Age, ethnicity or race, education level, income, and place of residence are key factors contributing to digital health inequities.  Aging can lead to declines in sensory perception, cognitive function, and physical ability.  A lack of social support can push vulnerable groups into digital exclusion.  Poor usability and operational complexity can create additional barriers to access and use. | Enhance your health self-efficacy, strengthen your confidence in self-management, improve health outcomes, and safeguard your health rights. |
| Song QC et al.(48) | 2025 | China | Review | Infrastructure gaps and urban-rural disparities.  Social and economic status can lead to unequal access to resources.  Physical decline may affect the ability to operate digital tools.  Trust in technology and concerns about information-related risks can hinder use.  A lack of age-friendly design and a mismatch between products and the needs of older adults can reduce usability.  A preference for in-person medical consultations may also limit the adoption of digital health services. | Internet penetration and the proportion of older internet users remain relatively low in rural areas.  Older adults in rural areas may be able to use only a limited number of basic smartphone functions.  Aging can lead to blurred vision, hearing loss, reduced physical coordination, and slower fine motor skills.  Without external support, older adults may be excluded from the digital health service system.  Medical and health apps, as well as online hospital services, have been slow to adopt age-friendly design for older users. | Data sharing can reduce the burden of home care and help divert pressure from offline medical resources.  It can improve health self-efficacy and proactive self-management, thereby enhancing health outcomes.  Services often prioritize efficiency while overlooking dignity and emotional needs, and technology empowerment may lead to value misalignment in practice. |
| Jiang YJ et al.(49) | 2024 | China | Qualitative | Socioeconomic status can make it difficult for older adults to access digital devices.  The older left-behind adults are, and the more likely they are to live alone, the less willing they may be to seek help from their children or others when using digital devices.  Small text on mobile phones, low call volume, and age-related visual or hearing decline can make digital devices difficult to use.  The large number of online advertisements can make it difficult for older adults to distinguish between genuine and misleading information. | Blurred vision and declining memory can make digital technology more difficult for older adults to use.  Older adults, especially the oldest age groups, may face greater difficulties in using digital technology.  Insufficient policy support, uneven urban-rural resource distribution, and products that do not meet the needs of older adults can all hinder use.  Fear of technology and concerns about privacy and security may also reduce willingness to use it. | Safeguard the health of older adults and promote health equity among the elderly population.  Left-behind older adults face even more severe social isolation. |
| Dong JK et al.(55) | 2025 | China | Conceptual framework | Limited access to technological resources.  Some older adults may not own smartphones or may not know how to use them.  Older adults often need repeated practice to master new technologies, and short-term assistance is usually insufficient to build lasting digital skills.  Fonts and buttons should be enlarged, and interfaces and operating steps should be simplified. | Urban-rural disparities.  A lack of willingness to learn and fixed mindsets.  Distrust of technology.  Empty-nest living arrangements and a lack of intergenerational support.  Insufficient motivation and initiative among companies to participate in governance.  Complicated operations and cumbersome interfaces. | Promote universal and equitable access to services in order to reduce digital health inequalities.  The digital divide can lead to a negative cycle affecting both physical and mental health. |
| Song X et al.(59) | 2025 | China | A systematic review and meta-analysis | Problems with interface design and usability.  Declining physical and cognitive functions. | Older adults with lower levels of education are more likely to develop a fear of technology.  Cognitive decline can further hinder the use of digital tools.  A lack of family support makes it difficult to address the challenges older adults face in using digital technologies.  Poor interface usability and complicated operations can also reduce accessibility and willingness to use these tools. | Manage your health information to improve your overall health. |
| Fang Z et al.(68) | 2024 | China | Quantitative | Expected health outcomes.  Self-efficacy | Low self-efficacy and distrust .  The digital divide in internet access. | Enhance older adults’ well-being and resilience to stress. Negatively affect older adults’ physical and mental health. |
| Cui YP et al.(44) | 2024 | China | Quantitative | Urban-rural differences, as well as disparities in regional resources and infrastructure.  Differences in socioeconomic status.  Family resources and intergenerational support.  Learning ability.  Physical and cognitive decline.  Limited ability to evaluate health information. | Age-related declines in physical functioning may increase the difficulty older adults experience in using digital technologies.  Older adults may demonstrate relatively limited learning capacity when adapting to new technologies.  Family guidance and support can facilitate older adults’ acquisition of digital skills and accelerate their adaptation to digital technologies.  Insufficient social support may contribute to digital exclusion among older adults. | Promoting the downward allocation of resources to reduce digital exclusion and narrow health disparities. |
| Sarah Nouri et al.(45) | 2020 | USA | Mixed-methods | Limited access to information channels.  Limited digital literacy. | Inadequate infrastructure.  Insufficient policy support.  Operational complexity. | Promote universal and equitable access to services in order to reduce digital health inequalities. |
| Fang ML et al.(50) | 2018 | Canada | Scoping reviews | Limited or no internet access .  Cost as a major barrier to the adoption and continued use of eHealth.  Lower eHealth use among older adults with low socioeconomic status despite interest in technology .  Vision and functional impairments as barriers to accessing and using eHealth systems.  Lower willingness and intention to access and use eHealth services among individuals with lower educational attainment. Feeling overwhelmed by the large volume of online information.  Improved accessibility through touchscreens, voice recognition, and larger, clearer fonts. | Differences in age, gender, ethnicity, place of residence, and educational attainment.  Physical and cognitive impairments .  The need for social support to promote training, practice, and encouragement.  Lagging age-friendly adaptations. | Improving health outcomes among older adults.  Social participation among older adults excluded by digital technology may decline accordingly |
| Arsenijevic, J et al.(66) | 2020 | Netherlands. | Systematic literature review and meta-analysis | Lack of digital literacy and limited comprehension skills.  Distrust of technology. Inadequate alignment between content and design. | Design that facilitates usability | Improve health outcomes and enhance quality of life. |
| Batsis, J. A et al.(46) | 2019 | USA | A systematic review | Urban–rural disparities in infrastructure development.  Renovations to enhance age-friendly features. | Differences in age, gender, ethnicity, place of residence, educational attainment, income, and social status.  Physical and cognitive impairments.  Inadequate age-friendly design and operational complexity. | NR |
| Sinabell, I et al.(60) | 2024 | Australia | A systematic review | Older adults require more time to complete tasks and read instructions, and they may forget specific task requirements.  Due to age-related declines in functional abilities, operational errors may be more difficult for older adults to manage.  Mentor support can facilitate older adults’ learning of new systems. Finger-based input may be challenging for older adults. | Physical and cognitive impairments.  Social support.  User-centered design. | Ageing in place. |
| Karlsen, C et al.(65) | 2017 | Norway | A qualitative systematic review | Autonomy.  Older adults experience a sense of misalignment between technology and their needs. | Physical and cognitive impairments.  User-centered design based on user needs.  Physical and cognitive impairments. | Achieve aging at home. |
| Matthew-Maich, N et al.(69) | 2016 | Canada | A scoping review | Cognitive impairment.  Variations in levels of technological literacy.  Users are willing to overcome barriers to use when the perceived value is high.  Limiting the navigation interface to two levels or fewer. | Cognitive impairments.  Widespread uncertainty in policies and management systems.  Mobile devices equipped with large touchscreens and large virtual buttons; voice input functionality. | Reduce the burden of home caregiving. |
| Zhao YC et al.(61) | 2022 | China | Systematic scoping review | Low self-efficacy.  Visual impairment.  Inappropriate font size.  Text-dense content with insufficient visual support. | User-centered design.  Privacy concerns.  Low educational attainment and limited health/digital literacy.  Physical disabilities. | Reduce older adults’ willingness to learn and adopt new digital technologies |
| Gordon, N. P et al.(47) | 2016 | USA | Quantitative | Among individuals without home internet access, approximately 34.9% reported cost as the primary barrier.  Individuals with lower levels of educational attainment are generally less likely to use patient portals.  Limited digital literacy.  Low self-efficacy.  A preference for telephone communication over secure messaging. | Differences in age, gender, ethnicity, educational attainment, and income.  Limited experience with computers and the internet.  Older adults require assistance from others when accessing the internet.  Privacy concerns | Reduce older adults’ willingness to learn and adopt new digital technologies |
| Smith, S. G et al.(70) | 2015 | UK | Quantitative | Patients with sufficient health literacy are more likely to use messaging features. | Differences in age, gender, ethnicity, place of residence, and educational attainment.  Social support.  Internet connectivity. | Improve health outcomes and enhance patient satisfaction. |
| Luo, J et al.(51) | 2024 | China | Quantitative | Simplify the design to reduce users’ learning costs by adopting features such as larger typography and a clearer layout, while ensuring that operational logic aligns with users’ established habits.  Self-efficacy. | The use of larger font sizes can help reduce the learning burden.  Factors such as cognitive decline, diminished learning capacity, and physical conditions including impaired vision and hearing should also be taken into consideration. | It interferes with the day-to-day management of chronic illnesses. |
| Tanaka, M et al.(64) | 2024 | Japan | Qualitative | Elderly participants felt cost and technical issues could be barriers to using monitoring devices.  Many of the elderly participants were interested in using monitoring devices at home, particularly if not complicated. | A fixed mindset may hinder their willingness to adopt new technologies.  Impairments in vision and hearing may make the use of small-screen devices less convenient.  Declines in physical endurance and cognitive functioning may further limit effective use. Concerns about privacy may also affect their willingness to engage with such technologies. | It can improve overall health outcomes among older adults.  Excessive reliance on such technologies may lead to social isolation and reduced social participation. |
| Foster, M et al.(67) | 2022 | USA | Qualitative | Sensory impairments, cognitive changes, arthritis, and vision impairments.  Operational difficulties and the burden associated with use. | Physical and cognitive dysfunction.  The need for social support .  Privacy concerns. | Optimize disease management and enhance overall health outcomes.  Some mHealth products overly pursue functional simplification and operational efficiency, ignore the psychological feelings, emotional needs and personalized demands of the elderly. |
| Vergouw, J. W et al.(71) | 2020 | Netherlands | Qualitative | I am not familiar with online services.  I prefer to schedule appointments by telephone and to communicate with physicians in person.  I do not perceive the app as being useful.  I require a larger font size. | When using the app to purchase medication online, I am concerned about making errors.  When using the app to purchase medication online, I am concerned about making errors.  Participants reported that it was difficult to seek assistance from others whenever needed.  Excessive page hierarchy makes the operation process complex.  Participants experienced difficulties during the login process. | NR |
| Jiang, Y et al.(62) | 2022 | China | Qualitative | Dependence on adult children.  Dependence on adult children.  Although older adults are taught to use relevant platforms for chronic disease management, some learn slowly and forget quickly.  Participants expressed doubts about the accuracy of online consultation results.  I am unwilling to use online rehabilitation services because I am concerned that using them may compromise my personal privacy.  I was concerned that I would not be able to learn how to use a smartphone, so I gave up.  Communication by phone creates a sense of distance; people tend to prefer face-to-face interaction.  Older adults are willing to learn to use such services only when they are simple to operate. | Differences in educational attainment.  Physical and cognitive impairments.  Peer support and assistance.  Whenever I encounter difficulties in using a device, my son teaches me how to operate it.  Concerns about online fraud and fraudulent platforms make me reluctant to try online health services. | Encourage older adults to participate actively and rationally in chronic disease management.  Exposure to negative health information online may cause psychological discomfort. |
| Portz, J. D et al.(56) | 2019 | USA | Qualitative | Poor economic conditions and low levels of educational attainment.  Inadequate broadband infrastructure in rural areas.  Difficulty reading small text; large font sizes are readable, whereas small font sizes are difficult to see clearly.  Concerns about and fear of digital technology.  Older adults are more accustomed to traditional modes of accessing medical care, such as making appointments by telephone, attending in-person consultations, and obtaining medication offline. | Age, educational attainment, and income disparities.  Large text is legible, whereas small text is difficult to read, and the interface color scheme is not suitable.  Caregivers and family members can help alleviate older adults’ technology-related anxiety.  There are malfunctions in the system backend.  Privacy concerns. | Assist older adults in carrying out health management. |
| Man, R. E. K et al.(54) | 2025 | Singapore | Qualitative | Access to relevant channels is limited, and the cost of acquisition remains high.  There is a widespread issue of low levels of digital literacy.  Many individuals are slow in using electronic devices and encounter difficulties in becoming familiar with their operation.  There are concerns regarding the accuracy of remote consultations, particularly the risk that reliance solely on verbal descriptions may result in diagnostic errors. | Disparities in age, gender, ethnicity, income, educational attainment, and the use of smart devices.  Physical and cognitive impairments.  Trust in technology.  A societal service orientation that remains inclined toward traditional models.  Operational complexity. | Reduce the risk of cross-infection and optimize healthcare service delivery.  The vicious cycle of physical and mental health problems. |
| Jaana, M et al.(52) | 2025 | Canada | Quantitative | Compared with people living in rural areas, those in suburban or urban areas have consistently used health apps and searched for health information online more frequently.  People with limited financial resources or insurance coverage are the most likely to be digitally excluded.  People with postgraduate degrees show greater interest in using internet technology.  Even with limited experience, they are still willing to use digital tools. | A lack of policies specifically targeted at resource-constrained older adults.  A lack of motivation to use digital technologies, along with limited trust in them.  Differences in age, place of residence, educational attainment, and income. | Improve health outcomes.  Excessive online searching for health information can easily lead to psychological distress and interfere with appropriate health-related judgment and behavior. |
| Liu, N et al.(63) | 2021 | Australia | A systematic review | With advancing age, declines in visual and auditory function, manual dexterity, and information-processing capacity may occur.  Information should be presented in multiple formats, including text, images, and videos.  Users should be allowed to customize font and text settings. | The application should provide audio-based alternatives to reduce the burden on older adults with visual impairments. For older adults with motor impairments, text input may be challenging. | Improve health outcomes. |
| Kampmeijer, R et al(57) | 2016 | Netherlands | A systematic literature review | The monetary costs of use are too high.  Older adults often lack the necessary skills to operate eHealth and mHealth devices.  Instructions for operating these devices, as well as the information provided, are often unclear, which can easily lead to misunderstandings and reflects a lack of adequate guidance.  When electronic devices are simple to operate and easy to use, older adults are more willing to continue using them. | Differences in educational attainment and age.  Lack of motivation and support are common factors that hinder older adults’ sustained use of eHealth and mHealth interventions.  Barriers also arise from inadequate policies and reimbursement mechanisms.  Technical problems, such as network failures and device malfunctions, are common.  Information is often difficult to understand, and device instructions are unclear. | Limited internet use may reduce social interaction and increase feelings of loneliness.  Improve health outcomes among older adults. |
| Lee, J et al(53) | 2022 | Korea | Quantitative | Internet use is particularly low among groups with lower educational attainment and limited economic resources.  It is necessary to provide educational programs for older adults to help them develop the skills needed to access and evaluate online information and services. | Age, educational attainment, and income disparities.  The spread of online misinformation and false content affects older adults’ acceptance. | Promoting health equity in the digital era.  Underutilization of digital health technologies may reduce social interaction and increase feelings of loneliness. |
| Christensen, L. F et al.(58) | 2020 | Denmark | A systematic review | Barriers to treatment include limited resources and the long distances patients must travel to reach treatment facilities.  Patients and physicians were initially skeptical and fearful, but also curious.  Older adults may experience sensory impairments and may be concerned about their unfamiliarity with telepsychiatry equipment, making them reluctant to use video consultations. | Lack of infrastructure.  Physical and cognitive impairments. | NR |

NR: not reported.

**The specific search strategies are detailed:**

A total of 10,694 articles were retrieved, of which 4,102 remained after deduplication using EndNote X9. Among the 4,102 publications, most focus on general digital health literacy, digital medical technology development, macro-level reviews of health policy, and smart medical device development.Based on this, a screening of titles and abstracts was conducted for each article. Consequently, 3,558 articles were excluded because they consisted solely of introductions to digital products, policy overviews without empirical data, conference abstracts, or popular science reviews. This process left 544 articles for further analysis.Subsequently, the remaining 544 articles underwent a first-round screening. Of these, 45 articles were excluded, including 6 that introduced platform websites, 14 published in languages other than Chinese or English, and 25 for which the full text was unavailable. This process left 499 articles after the initial screening.After conducting a full-text review, a total of 461 articles were excluded. This included 13 articles identified as low-quality clinical trial reviews, 392 articles that discussed general digital health without addressing equity issues, and 56 articles whose research did not focus on older adults. Because some relevant studies were not captured in the initial search due to variations in titles or keywords, an additional six papers were identified through reference screening during the full-text review stage. These supplementary papers primarily addressed theoretical frameworks and key factors influencing digital health equity among older adults. In total, 44 eligible studies were included, comprising 7 Chinese-language studies and 37 English-language studies.


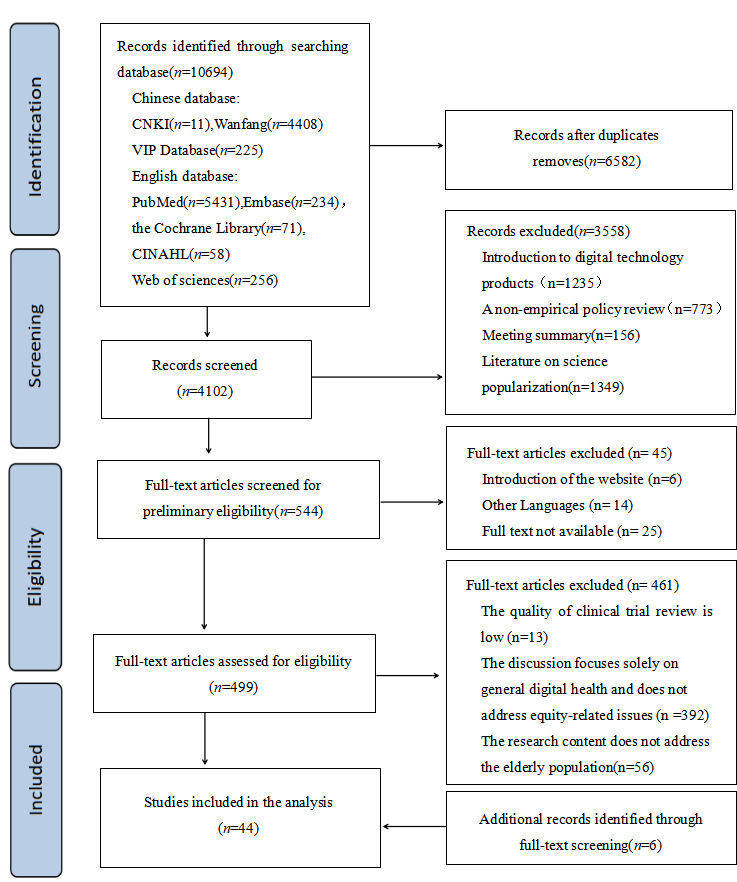


FIGURE 1 Flow diagram of literature search and selection.

| **Database** | **Search fields** | **Boolean operators** | **Filters** | **Search dates** | **Language limits** |
| --- | --- | --- | --- | --- | --- |
| CNKI | Topic | (Elderly OR Senior OR Advanced Age OR Aging Population) AND (Digital Health OR Digital Health Equity OR e-Health OR Mobile Health OR Telemedicine OR Smart Health OR Equity OR Health Inequality OR Digital Divide) | No filters applied | The retrieval period spanned from database inception to November 30, 2025 | Chinese |
| Wanfang Data | Title / Keywords | Title or keywords: (elderly or senior citizens or advanced age or aging) and title or keywords: (digital health or digital health equity or e-health or mobile health or telemedicine or smart health or equity or health equity or digital health inequality or digital divide) | No filters applied | The retrieval period spanned from database inception to November 30, 2025 | Chinese |
| VIP Database | Title / Keywords | M = (elderly OR seniors OR advanced age OR aging) AND M = (digital health OR digital health equity OR e-health OR mobile health OR telemedicine OR smart health OR equity OR health equity OR digital health inequality OR digital divide) | No filters applied | The retrieval period spanned from database inception to November 30, 2025 | Chinese |
| PubMed | Title/Abstract | (((((((Digital Health[MeSH Terms])OR(digital health[Title/Abstract]))OR(eHealth[Title/Abstract]))OR(mHealth[Title/Abstract]))OR(telemedicine[Title/Abstract]))OR(((Health Equity[MeSH Terms])OR(health inequity[Title/Abstract]))OR(health equity[Title/Abstract])))OR(digital health equity[Title/Abstract]))AND((((Aged[MeSH Terms])OR(elderly[Title/Abstract]))OR(older adult*[Title/Abstract]))OR(senior*[Title/Abstract])) | No filters applied | The retrieval period spanned from database inception to November 30, 2025 | English |
| Embase | All fields | ('aged'/exp OR elderly OR "older adult" OR "older people" OR "senior citizen" OR geriatric OR "ageing population") AND ('digital health'/exp OR "e-health" OR "electronic health" OR "mobile health" OR "m-health" OR "digital healthcare" OR "online health" OR "internet health") AND ('health equity'/exp OR "health equality" OR "health disparity" OR "health inequity" OR "health fairness" OR "social justice in health" OR "digital divide") | No filters applied | The retrieval period spanned from database inception to November 30, 2025 | English |
| Web of Sciences | Topic | (aged OR elderly OR older adults OR elder adults OR senior citizens) AND (digital health equity OR digital health OR telemedicine OR mHealth OR eHealth OR health inequity) | No filters applied | The retrieval period spanned from database inception to November 30, 2025 | English |
| CINAHL | Title/Abstract/Subject Terms | (MH "Aged+" OR elderly OR "older adult" OR "older people" OR senior OR geriatric OR "ageing population") AND (MH "Health Equity" OR "health equality" OR "health disparity" OR "health inequity" OR "health fairness") AND ("digital health" OR "e-health" OR "electronic health" OR "m-health" OR "mobile health" OR "digital healthcare" OR "online health") AND (TI OR AB OR MH) | No filters applied | The retrieval period spanned from database inception to November 30, 2025 | English |
| the Cochrane Library | Title/Abstract/Keywords | (MeSH descriptor: [Aged] explode all trees OR elderly OR "older adult" OR "older people" OR senior OR geriatric OR "ageing population") AND (MeSH descriptor: [Health Equity] explode all trees OR "health equality" OR "health disparity" OR "health inequity" OR "health fairness") AND ("digital health" OR "e-health" OR "electronic health" OR "m-health" OR "mobile health" OR "digital healthcare" OR "online health") [ti,ab,kw] | No filters applied | The retrieval period spanned from database inception to November 30, 2025 | English |
